# Molecular analysis of the UV-inducible pili operon from *Sulfolobus acidocaldarius*

**DOI:** 10.1002/mbo3.128

**Published:** 2013-09-19

**Authors:** Marleen van Wolferen, Małgorzata Ajon, Arnold J M Driessen, Sonja-Verena Albers

**Affiliations:** 1 Molecular Biology of Archaea, Max Planck Institute for terrestrial MicrobiologyKarl-von-Frisch-Strasse 10, 35043, Marburg, Germany; 2 Department of Molecular Microbiology, Groningen Biomolecular Sciences and Biotechnology Institute, University of GroningenNijenborgh 7, NL-9747 AG, Groningen, The Netherlands

**Keywords:** Archaea, conjugation, DNA exchange, type IV pili

## Abstract

Upon ultraviolet (UV) stress, hyperthermophilic *Sulfolobus* species show a highly induced transcription of a gene cluster responsible for pili biogenesis: the UV-inducible pili operon (*ups* operon). This operon is involved in UV-induced pili assembly, cellular aggregation, and subsequent DNA exchange between cells. As the system increases the fitness of *Sulfolobus* cells after UV light exposure, we assume that transfer of DNA takes place in order to repair UV-induced DNA damages via homologous recombination. Here, we studied all genes present in the *ups* cluster via gene deletion analysis with a focus on UpsX, a protein that shows no identifiable functional domains. UspX does not seem to be structurally essential for UV-induced pili formation and cellular aggregation, but appears to be important for efficient DNA transfer. In addition, we could show that pilin subunits UpsA and UpsB probably both function as major pilin subunits in the ups pili.

## Introduction

Upon ultraviolet (UV) stress *Sulfolobus* species show a high upregulation of a gene cluster encoding proteins responsible for formation of type IV pili (T4P) (Fröls et al. 2007; Götz et al. 2007): the *ups* operon (UV-inducible pili operon of *Sulfolobus*) (Fröls et al. 2008). Indeed, when analyzed by transmission electron microscopy (TEM), a large number of pili could be observed on the surface of UV-stressed *Sulfolobus* cells from different species (Ajon et al. 2011). An additional phenotypic characteristic of UV-stressed *Sulfolobus* cells is the formation of large cellular aggregates (Fröls et al. 2008), which was shown to be mediated by ups pili in a species-specific manner (Ajon et al. 2011). Moreover, *Sulfolobus* cells can exchange chromosomal DNA in a pili-dependent manner which was shown to increase cellular fitness under UV stress (Ajon et al. 2011). Transfer of DNA therefore probably plays a role in repair of double-strand breaks (DSBs) caused by UV radiation as was proposed in Ajon et al. (2011). Because the aggregation was also shown to be inducible by the DNA strand-break-inducing agent bleomycin, the first trigger for pili formation and subsequent aggregation is thought to be the sensing of DSBs in the DNA (Fröls et al. 2008). The mechanism behind this process remains unknown. DNA exchange mechanisms among hyperthermophiles for repair of DNA have been described in more detail by van Wolferen et al. (2013).

In other organisms, T4P were also shown to mediate DNA transfer (Filloux 2010). Different studies showed that the pili facilitate the uptake of extracellular DNA in competent Gram-positive and Gram-negative bacteria. The exact role of T4P in competence is still not well understood, but their presence is essential for successful DNA uptake (reviewed in Krüger and Stingl 2011). It has been suggested that DNA is brought close to the cell surface by binding to the pili that subsequently retract. A recent study for the first time reported the binding of a pilin subunit (minor pilin ComP) of competent *Neisseria meningitidis* to specific sequences of self-DNA suggesting that pili indeed bring DNA to the cell surface (Cehovin et al. 2013). Besides having a role in competence, two examples are known in which T4P are involved in a conjugative system. First, the IncI1 conjugative plasmid R64 in *Escherichia coli* that carries the *pil* genes (Kim and Komano 1997; Yoshida et al. 1999; Komano et al. 2000), and second, PAPI-1 DNA which conjugates between *Pseudomonas* species and also comprises *pil* genes. The latter is located on a pathogenicity island that has been obtained via horizontal gene transfer (Carter et al. 2010). A role of T4P in cellular chromosomal exchange and subsequent repair of damaged DNA has never been shown before. Moreover, unlike the described bacterial T4P, the archaeal *ups* genes are not present on a conjugative plasmid or acquired via horizontal gene transfer.

The UV-induced pili system was initially studied in *S. solfataricus* (Fröls et al. 2008; Ajon et al. 2011), but because of the availability of genetic tools for manipulating the *Sulfolobus acidocaldarius* genome (Wagner et al. 2009, 2012) we switched to the latter organism. There is a high conservation and functional similarity among all sequenced *Sulfolobus* species. The *ups* operon encodes five proteins: UpsX, a hypothetical protein with no conserved regions; UpsE, an ATPase; UpsF an integral membrane protein; and UpsA and UpsB, two putative pilin subunits containing class III signal peptides.

Here, we have analyzed the different roles of the genes in the *ups* cluster. The secretion ATPase UpsE and the membrane protein UpsF are, as expected, essential for the formation of ups pili. Interestingly, UpsX is not essential for pili formation, but the deletion of its gene resulted in decreased DNA exchange suggesting a role in DNA transfer. Pilin subunits UpsA and B are essential for cellular aggregation. However, as deletion mutants of either of the subunits still formed pili, it appears that a mixed structure is essential for cellular aggregation.

## Material and Methods

### Culture conditions

*Sulfolobus acidocaldarius* strains MW001, MR31, and JDS183 and derived mutants were grown aerobically at 78°C in basic Brock medium (Brock et al. 1972), supplemented with 0.1% NZ amine AS (Sigma, Munich, Germany), 0.2% dextrin, and 20 μg/mL uracil, and adjusted to pH 3.5 with sulfuric acid. For solid media the medium was supplemented with 1.5% gelrite. Plates were incubated for 5–6 days at 78°C. *E. coli–*competent cells DH5α and ER1821 (NEB, Frankfurt am Main, Germany), used for, respectively, cloning and methylation of plasmid DNA, were grown in Lysogeny broth medium (10 g/L tryptone; 5 g/L yeast extract; 10 g/L NaCl) at 37°C supplemented with the appropriate antibiotics. Growth of cells was monitored by optical density measurements at 600 nm.

### Ultraviolet treatment, aggregation assays

Ultraviolet light treatment was performed as described in Fröls et al. (2008). Ten mL culture (OD_600_ 0.2–0.3) was treated with a UV dose of 75 J/m^2^ (254 nm, UV crosslinker; Spectroline, Westbury, NY) in a plastic petri dish. Subsequently cultures were incubated at 78°C for 3 h. Samples taken at different time points were analyzed with phase contrast microscopy, survival rate assays, and electron microscopy. To quantify aggregated cells after induction with UV, 5 μL of cell culture (diluted to OD 0.2) was spotted on a microscope slide covered with a thin layer of 1% agarose in Brock minimal medium. A coverslip was added when the drop had dried. Cells were visualized with phase contrast microscopy. Free and aggregated cells (≥3) were counted for at least three fields per strain using ImageJ cell counter (NIH, Bethesda, MD). Percentages of cells in aggregates were subsequently calculated.

### Deleting/tagging genes in *S. acidocaldarius*

To construct deletion and gene replacement strains, up- and downstream flanking areas of the genomic regions of interest (∼600 bp) were amplified with primers listed in Table S1. Primers were designed according to the genomic sequence of *Sulfolobus acidocaldarius* DSM639. Overlap polymerase chain reaction (PCR) was performed to connect the up- and downstream fragments (Wagner et al. 2012). The PCR product was subsequently cloned into pSVA406, carrying an ampicillin resistance gene, which resulted in the plasmids summarized in Table 1. The plasmids were methylated in *E. coli* ER1821-containing pM.EsaBC4I (NEB) (Kurosawa and Grogan 2005) and transformed into *S. acidocaldarius* MW001/MR31 (Wagner et al. 2012). Integrants were selected on plates lacking uracil and grown in 24-well plates for 2 days in the same medium. Subsequently cultures were plated and grown for 5 days on second selection plates containing uracil and 100 μg/mL 5-fluoroorotic acid to select for clones in which the plasmid looped out by homologous recombination. Obtained colonies were tested by PCR for successful deletion/replacement of the genes. Correctness of strains was confirmed by DNA sequencing. Strains that were made during this study are listed in Table 2.

**Table 1:** Plasmids used during this study

Plasmid	Description	Source/Reference
pMA08	Deletion plasmid for Δ*upsX*+promoter region *(*Δ*saci1493,* −40 bp*)*	This study
pSVA406	Backbone of deletion plasmids	(Wagner et al. 2012)
pSVA180	Deletion plasmid for Δ*aapF (*Δ*saci2318)*	A. Gosh and S.V. Albers, unpubl. data
pSVA329	Deletion plasmid for Δ*flaI* (*saci1173* Δbp 1–672)	A. Gosh and S.V. Albers, unpubl. data
pSVA1819	Deletion plasmid for Δ*upsX (*Δ*saci1493)*	This study
pSVA1801	Deletion plasmid for Δ*upsA (*Δ*saci1496)*	This study
pSVA1802	Deletion plasmid for Δ*upsB (*Δ*saci1496b)*	This study
pSVA1805	Deletion plasmid for Δ*upsF (*Δ*saci1495)*	This study
pSVA1832	Plasmid to add C-term HA tag on *upsX (saci1493)*	This study

**Table 2:** Strains used during this study

Strain	Background strain	Genotype	Source/Reference
MR31 (wt1)	*S. acidocaldarius* DSM639	Δ*pyrE* (Δbp 154–171)	Reilly and Grogan (2001)
JDS183 (wt2)	*S. acidocaldarius* DSM639	Δ*pyrE* (2xbp 44)	Grogan and Hansen (2003)
SA1 (Δ*upsE1*)	*S. acidocaldarius* MR31	Δ*upsE*+Δ*upsF*	Ajon et al. (2011)
DG253 (Δ*upsE2*)	*S. acidocaldarius* SA1	Δ*upsE*+Δ*upsF,* Δ*pyrE* (Δbp 335)	Ajon et al. (2011)
GA06	*S. acidocaldarius* MR31	Δ*upsX*-40 bp	This study
GA07	*S. acidocaldarius* MR31	Δ*upsX*	This study
GA09 (Δ*upsX)*	*S. acidocaldarius* GA07	Δ*upsX* Δ*pyrE* (Δbp 28)	This study
MW001	*S. acidocaldarius* DSM639	Δ*pyrE* (Δbp 91–412)	Wagner et al. (2012)
MW101	*S. acidocaldarius* MW001	*upsX*+ C-term HA	This study
MW106	*S. acidocaldarius* MW001	Δ*upsA*	This study
MW107	*S. acidocaldarius* MW001	Δ*upsB*	This study
MW109	*S. acidocaldarius* MW001	Δ*upsE*	Wagner et al. (2012)
MW110	*S. acidocaldarius* MW001	Δ*upsF*	This study
MW115	*S. acidocaldarius* MW001	Δ*upsX*	This study
MW138	*S. acidocaldarius* MW501	Δ*upsA,* Δ*flaI* (Δbp 1–672)*,* Δ*aapF*	This study
MW140	*S. acidocaldarius* MW501	Δ*upsB,* Δ*flaI* (Δbp 1–672)*,* Δ*aapF*	This study
MW501	*S. acidocaldarius* MW001	Δ*flaI* (Δbp 1–672)*,* Δ*aapF*	A. Gosh and S.V. Albers, unpubl. data

### qPCR and operon mapping on *S. acidocaldarius* cDNA

To compare expression of the *ups* operon from different strains (UV/not-UV induced), RNA was isolated from 10 mL cultures (from MW001 and mutants, Table 2) using TriFast™ (Peqlab, Erlangen, Germany). DNA was subsequently degraded by incubating the RNA with DNAseI (RNAse free, Fermentas, St. Leon-Rot, Germany) according to the manufacturer's protocol. Proper DNA degradation was confirmed by performing a PCR with primer pair 2033 + 2087 (Table S1) on the RNA. cDNA synthesis was performed on 1 μg of RNA with the First Strand cDNA Synthesis Kit (Fermentas). Random primers were used and the manufacturer's protocol was followed. Quantitative PCR (qPCR) was performed using the Maxima SYBR Green/ROX qPCR master mix. qPCR primers were designed for *saci1493–saci1496b* (*upsX-upsB*), they have a melting temperature around 60°C and give a product of 80–150 bp in length (primers 2073–2082, Table S1). As a control, primers for an *lrs14* gene that was not found to be differentially expressed after induction with UV in microarray studies were used. Control qPCR was performed according to the manufacturer's instructions. The obtained CT values were used to compare non-UV–induced with UV-induced expression of the tested genes. Moreover, expression was compared between MW001 and deletion strains. Differences in expression were displayed as log^2^ folds.

To determine whether the genes in the *ups* gene cluster are present as an operon, isolated cDNA of UV-induced *S. acidocaldarius* MW001 was used to amplify intergenic regions between *upsX* and *E*; *E* and *F*; *F* and *A*; and *A* and *B* using primer pairs: 2073 + 2076; 2020 + 2013; 2077 + 2080; and 2038 + 2082, respectively (Table S1).

### DNA transfer assays

DNA transfer between *S. acidocaldarius* cells was assayed by selecting prototrophic (*pyr*^*+*^) recombinants of two *pyrE* mutant strains. The *ups*^+^ *pyrE* strains were MR31 (wt1) and JDS183 (wt2). Strain MR31 has an 18-bp deletion in *pyrE* (nt 154–171). Strain JDS183 contains a frame shift mutation (duplication of T) at nt 44. The Δ*upsE* strains were SA1 (Δ*upsE1*) and DG253 (Δ*upsE2*). SA1 was derived from background strain MR31, and DG253 contains a transition mutation (A–G) at *pyrE* nt 335. The Δ*upsX* strain was GA09, which contains a frameshift mutation in *pyrE* (Δbp 28, see Table 2). Liquid cultures were grown at 78°C and harvested at OD_600_ 0.15–0.35. Pellets were resuspended to a cell density of about 2 × 108 cells/mL. UV irradiation was performed as described before (Ajon et al. 2011). Recombination was assayed by spreading a mixture with 50 μL of each of two suspensions on selective plates without uracil. Plates were incubated for 5–6 days at 78°C as was described previously (Ajon et al. 2011). For each experiment the results were normalized, taking mixture wt1(UV) × wt2 (C) as 100%.

### Electron microscopy analysis

Ultraviolet-induced pili in *S. acidocaldarius* cells and derived mutants were observed with TEM. Specimens were negatively stained with 2% uranyl acetate on carbon-coated copper grids. Microscopy was performed with a Philips CM10 electron microscopy operated at 120 kV. Images were recorded using a Gatan 4K CCD camera at different magnifications.

### Western blot analysis of HA-tagged UpsX

Ultraviolet irradiation on MW001 and MW101 was performed as described before above. Subsequently 20 mL of control and UV-treated samples were harvested at 0, 1, 2, and 3 h time points and resuspended in 50 mmol/L 4-(2-hydroxyethyl)-1-piperazineethanesulfonic acid (HEPES), buffer pH 8, containing 150 mmol/L KCl. Cells were broken by 10 cycles of sonication. Unbroken cells were removed by low spin centrifugation (8000 rpm × 10 min, 4°C). Cytoplasmic and membrane fractions were obtained during ultracentrifugation step (70,000 rpm × 30 min, 4°C). Isolated membranes were solubilized in 50 mmol/L HEPES/150 mmol/L KCl buffer pH 8, supplemented with 1% Triton X-100 for 45 min at room temperature. All samples were run on a 12% sodium dodecyl sulfate polyacrylamide gel electrophoresis (SDS-PAGE) gel. Western blot was performed on polyvinylidene difluoride (PVDF) membrane, blocked overnight with 2% I-Block in PBST (150 mM NaCl; 20 mM Na_2_HPO_4_; 0.1% Tween; pH 7.4), and followed by incubation with 1:100 primary anti-HA antibodies (Sigma) and 1:30,000 secondary anti-Rabbit IgG-Alkaline Phosphatase antibodies (Sigma). Chemiluminescence signal was obtained by CDP-Star (Roche, Woerden, the Netherlands) on Roche Lumi-imager. Intensities of the bands were quantified using ImageJ.

## Results

### Bioinformatics and transcriptional analysis on the *ups* operon

**Figure 1:**
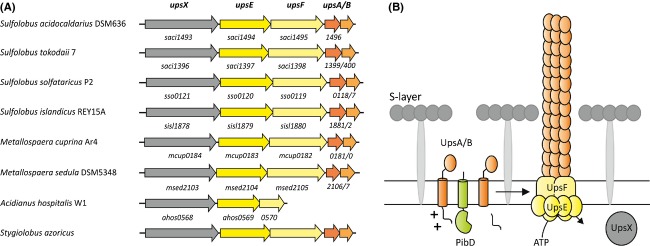
Schematic overview of the *ups* gene cluster in different Sulfolobales. The cluster encodes: UpsX, a protein with unknown function; UpsE, a secretion ATPase; UpsF, an integral membrane protein; and UpsA and B, two pilin subunits. As not all genes (especially the pilin subunits) were annotated properly, they were corrected by hand. Homology found using SyntTax (Oberto 2013) is indicated by similar colors. The relevant portion of the genomic DNA sequence of *Stygiolobus azoricus* was annotated manually (A. Wagner and S.-V. Albers, unpubl. data).

As described before (Fröls et al. 2008), the *ups* operon encodes five proteins that are together thought to build the type IV pilus (Fig. 1): UpsE, a secretion ATPase; UpsF, an integral membrane protein; UpsA and UpsB, two putative pilin subunits containing class III signal peptides; and UpsX, a protein with unknown function. UpsX is a predicted cytoplasmic protein, but in contrast to UpsE, F, A, and B, no specific domains could be predicted by BLAST (Altschul et al. 1990), SMART (Schultz et al. 1998), and HHpred (Söding 2005) (data not shown). An alignment of UpsX sequences from different Sulfolobales revealed several conserved regions and amino acids (Fig. S1). As the bioinformatics did not predict a possible UpsX function, it was subjected to further functional analysis.

**Figure 2:**
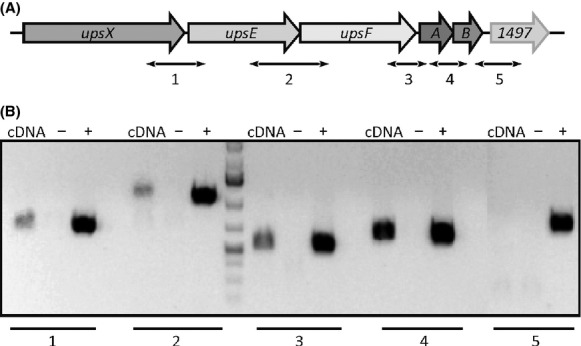
Operon mapping on the *ups* gene cluster of *Sulfolobus acidocaldarius*. (A) Gene organization of the *ups* gene locus and downstream gene *saci1497*. Black numbered arrows indicate the regions of interest for amplification. (B) RT-PCR on cDNA from *S. acidocaldarius* MW001 induced with UV. Genomic DNA was used as positive control (+) and RNA was treated with DNase as negative control (−).

**Figure 3:**
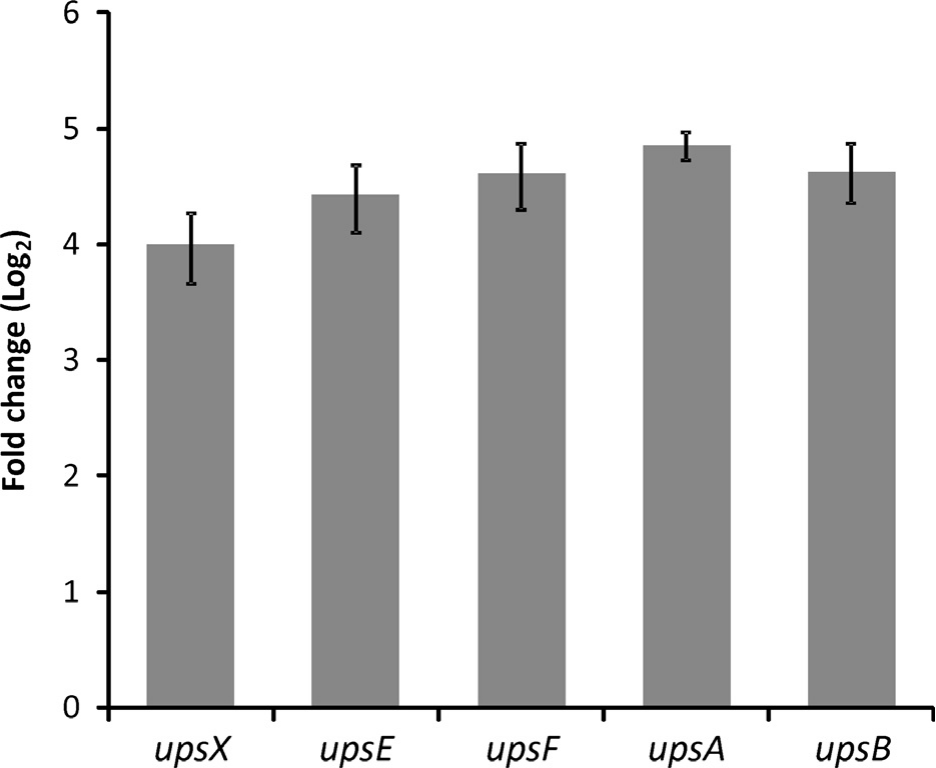
Transcriptional regulation of *ups* genes upon UV treatment. Change in transcription levels of genes in the *ups* operon in MW001 3 hours after UV treatment (75 J/m^2^) as measured by qRT-PCR. Differences are displayed as Log_2_ fold changes. Used primers are summarized in Table S1.

Synteny analysis (SyntTax; Oberto 2013) revealed that all sequenced Sulfolobales contain a *ups* operon (Fig. 1), including recently sequenced *Stygiolobus azoricus* (A. Wagner and S.-V. Albers, unpubl. data). However, as *Acidianus hospitalis,* member of the Sulfolobales, lacks genes encoding pilin subunits UpsA and UpsB, this species probably does not build functional ups pili. In addition, the membrane protein encoding *upsF* seems to be incomplete in this species. It is therefore likely that *A. hospitalis* lost part of its *ups* operon throughout evolution. Deep sequencing on *S. acidocaldarius* cDNA (O. Wurtzel, unpubl. data) revealed a transcriptional start site (TSS) in front of *upsX* (with ∼10,000 transcript reads)*,* and TSSs in front of the *upsE* (160 reads) and *upsA* (133 reads) genes. These results suggest a primary TSS in front of *upsX* and secondary TSSs in front of *upsE* and *upsA*. Operon mapping using RT-PCR indeed shows that transcripts of *upsX-B* are connected and therefore probably present as one long transcript (Fig. 2). This is confirmed by the observation that a deletion of both the *upsX* gene and its promoter (Δ*upsX -*40) leads to an unpiliated phenotype, whereas a clean deletion of *upsX* does not (Fig. 6). Interestingly, the deep sequencing data revealed several antisense TSSs in *upsB*, which might have a regulatory function. Importantly, these deep sequencing data show already transcription numbers without prior UV induction of the samples, indicating that the operon is expressed highly even without UV stress. The ups system therefore probably shows a high basal activity. Previous microarray studies showed an upregulation of the transcription of all *ups* genes with log_2_ folds of up to 3 (Fröls et al. 2007; Göötz et al. 2007). By means of qPCR, this induction could be confirmed showing even higher log_2_ folds of between 4 and 5 (Fig. 3).

### Deletion mutant analysis of genes in the *ups* operon

**Figure 4:**
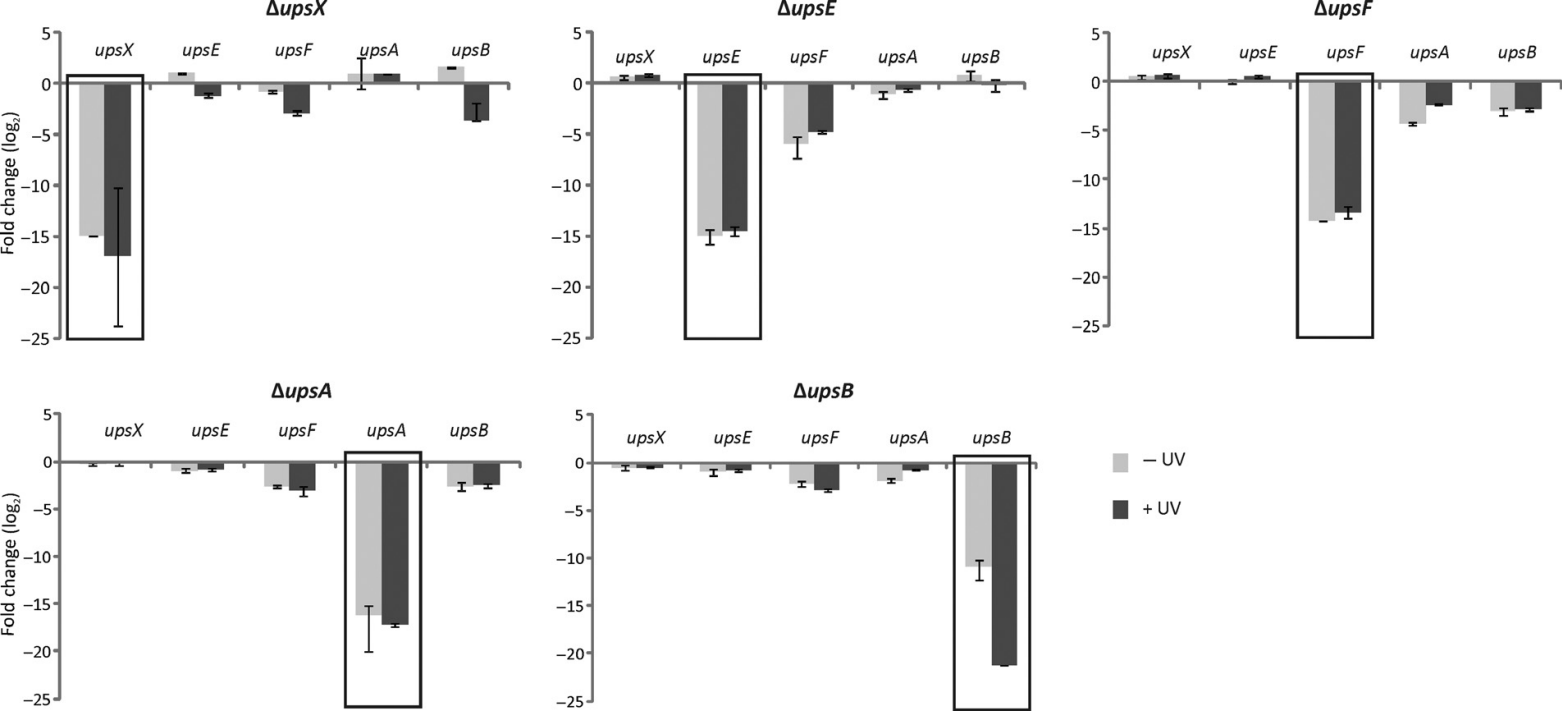
qPCR analysis comparing gene expression from *ups* mutants with that from wild-type *Sulfolobus acidocaldarius*. To show the effects of *ups* deletions on neighboring gene expression, RNA from deletion mutants, treated with or without UV (light and dark gray, respectively), was isolated and converted into cDNA. cDNA was subjected to qRT-PCR analysis using primers specific for indicated *ups* genes. Strains used were as follows: Δ*upsX* (MW115), Δ*upsE* (MW109), Δ*upsF* (MW110), Δ*upsA* (MW106), and Δ*upsB* (MW107). Shown are Log_2_ fold-change values in expression compared to MW001. Bars marked with a box are the deleted gene of the corresponding mutant. The means and standard deviations of biological replicates are shown.

**Figure 5:**
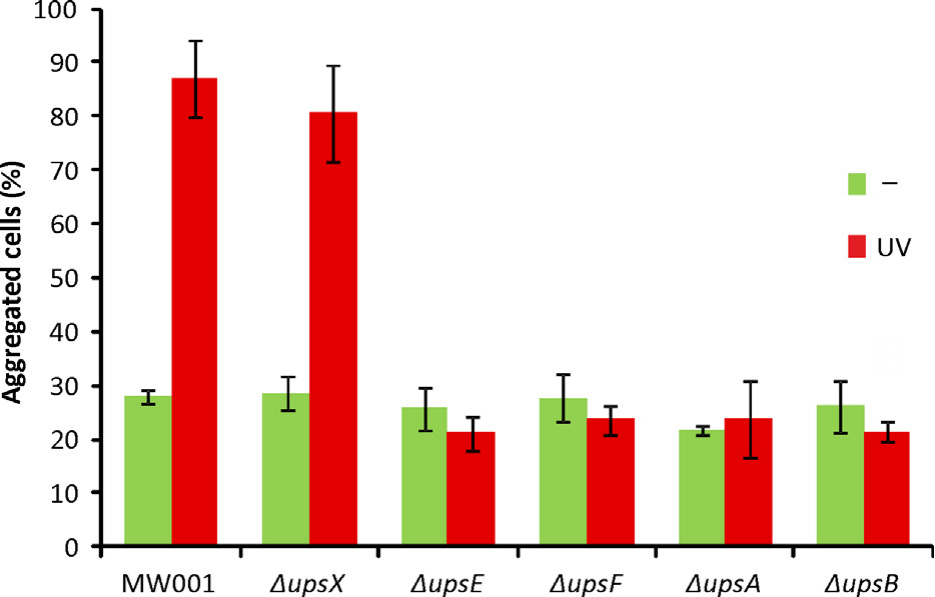
Aggregation assays with *ups* deletion strains. Light microscopy analysis of aggregation of the different *ups* deletion strains 3 h after induction with UV (75 J/m^2^). Strains used were as follows: Δ*upsX* (MW115), Δ*upsE* (MW109), Δ*upsF* (MW110), Δ*upsA* (MW106), and Δ*upsB* (MW107).

**Figure 6:**
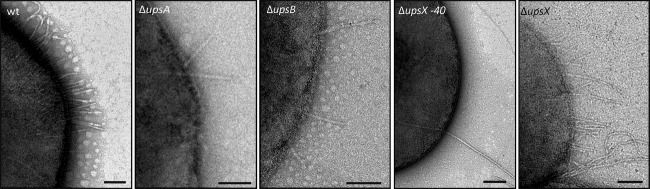
Transmission electron microscopy on UV-induced *Sulfolobus acidocaldarius* mutants. Pili formation of wild-type *S. acidocaldarius* cells (MR31), Δ*upsA* (MW138), Δ*upsB* (MW140), Δ*upsX* -40 (including promoter region, GA06), and Δ*upsX* (MW115). *Scale bar* 100 nm.

In order to obtain more insights in the individual roles of the genes in the *ups* operon, markerless deletion mutants were created as described previously (Wagner et al. 2012) (Table 2). The genotypes were confirmed by PCR and sequence analysis (data not shown). In addition, transcription levels of *ups* genes in the different deletion mutants were compared with those from wild-type cells by qPCR (Fig. 4). Generally, no strong polar effects could be observed on downstream genes of the deleted genes of interest. However, the deletion of *upsE* resulted in a lower transcription of *upsF*. Moreover, in a *upsF* mutant transcription of *upsA* and *upsB* was slightly reduced. The latter might possibly be due to the partial removal of the possible promoter region of *upsA* by the deletion of *upsF*. Growth curves and microscopy revealed wild-type growth and a normal cellular phenotype for all deletion mutants (data not shown). In deletion mutants of *upsE, F, A,* and *B,* no UV-induced aggregation could be observed (Fig. 5). This was to be expected as the secretion ATPase (UpsE), membrane protein (UpsF), and pilin subunits (UpsA/B) are all thought to be essential for pili formation. In line with this, no ups pili formation could be observed for the *upsE* and *F* deletion strains (Ajon et al. 2011; Fig. 6, and data not shown). As it is unknown if and how the two predicted pilin subunits UpsA and UpsB together build up one filament, we also looked at single-deletion mutants of *upsA* and *upsB*. To not confuse ups filaments with other surface structures (Henche et al. 2012; Jarrell and Albers 2012), these strains were made in an archaella- and aap (archaeal adhesive) pili-less background (MW501). When the single mutants of *upsA* and *upsB* were analyzed with EM, both strains showed pili formation, although at smaller numbers. These results suggest that both subunits are capable to form filaments, but that for efficient pili formation both genes are needed, likely resulting in mixed subunit pili.

Interestingly, the Δ*upsX* strain shows wild-type cellular aggregation upon UV treatment and also the ups pili are wild type like (Figs. 5 and 6). UpsX therefore does not seem to have an essential role in the pili formation or cellular recognition (aggregation).

### Chromosomal marker exchange

**Figure 7:**
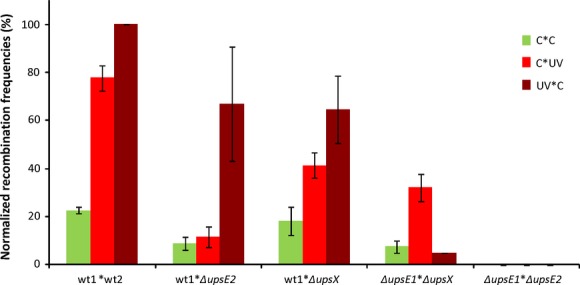
Recombination frequencies of mating experiments with *Sulfolobus acidocaldarius* wild-type and mutant strains. Two different strains treated with (UV) or without (C) UV were mixed in different combinations and plated on selective media. All strains contained mutations in the *pyr*E gene (involved in de novo uracil biosynthesis) located at different positions, such that recombination between two strains can restore the wild-type phenotype. Strains used were as follows: wt1 (MR31), wt2 (JDS183,) Δ*upsE1* (SA1)*,* Δ*upsE2* (DG253), and Δ*upsX* (GA09). Bars represent the average of three independent mating experiments each, every experiment was normalized to wt1 (UV) × wt2 (C) as 100%.

To study a putative role of UpsX in DNA exchange between *Sulfolobus* cells, DNA transfer assays were performed. Series of auxotrophic *ups/pyrE* double mutants were used in mating experiments (Table 2). Two parental strains were mixed together and upon exchange of chromosomal DNA, *pyrE* mutations could be restored via homologous recombination resulting in prototrophic colonies as described previously (Ajon et al. 2011). For each experiment the results were normalized, taking the mixture of wt1 (UV) × wt2 (C) as 100% (Fig. 7). A mixture of two ups wild-type strains resulted in the formation of recombinants (Fig. 7, wt1 × wt2, green bar), indicating DNA exchange between the two strains and confirming a high basal activity of the system, even without UV stress. Moreover, upon induction of one of the two strains with UV light, exchange of DNA increased four- to fivefold (Fig. 7, wt1 × wt2, red bars). A *upsE* deletion strain did not support transfer of DNA when treated with UV light (Fig. 7, wt1 × Δ*upsE2*, first red bar). Only when the wild-type strain (wt1) was induced with UV in this mixture, a significant increase in DNA exchange could be observed, showing that only one of the two strains needs to assemble pili for DNA exchange to occur. A mixture of two Δ*upsE* strains resulted in no DNA transfer (Δ*upsE1* × Δ*upsE2*). These results confirmed the previously observed essential function of ups pili for DNA exchange between *Sulfolobus* cells (Ajon et al. 2011). However, when mixing the wt2 with UV-induced Δ*upsX*, still a higher amount of recombinants were formed than without UV induction*,* although this increase was only about 50% of that of a wt1 × wt2 mixture. Similarly, a mixture of Δ*upsX* with Δ*upsE* resulted in a significantly lower increase in DNA exchange upon UV induction of the Δ*upsX* strain. These results imply that UpsX plays a role in the process of UV-induced DNA transfer between *Sulfolobus* cells, but that it is not essential. Possibly, UpsX plays a direct or indirect role in DNA transfer or processing of incoming/outgoing DNA.

### *In vivo* protein levels and localization of UpsX

**Figure 8:**
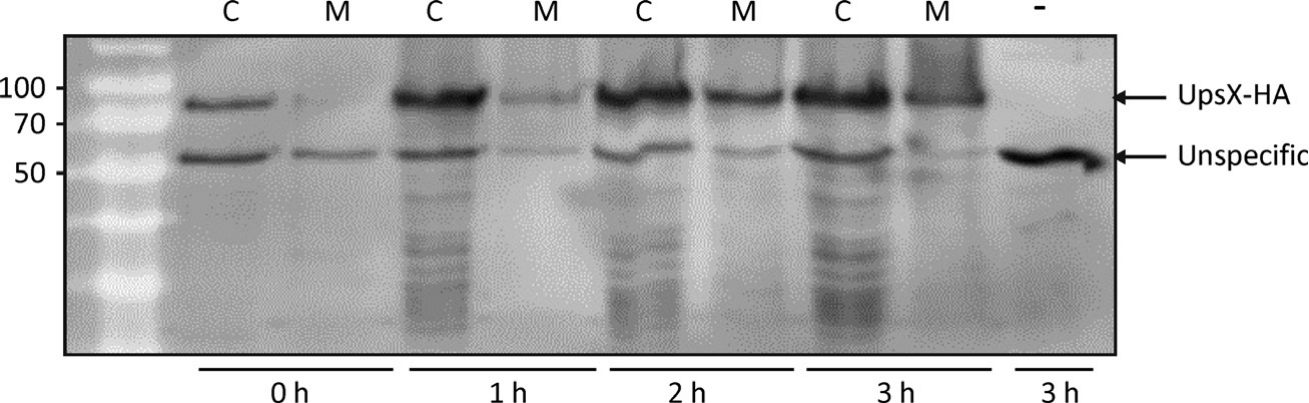
Time course of UpsX-HA expression upon UV treatment in MW101 cells. Membrane (M) and cytosol (C) fractions from MW101 (C-terminally HA-tagged UpsX) 0, 1, 2, and 3 h after UV induction were separated on SDS-PAGE. As a negative control, lysate from MW001 was used (−) showing a clear unspecific band, but no band at the size of UpsX-HA (79 kDa). Proteins were visualized by Western blotting using α-HA primary antibodies.

To localize UpsX in the cells, *S. acidocaldarius upsX* was genomically tagged with a C-terminal HA tag (MW101, Table 2). This strain was induced with UV and samples were taken 0, 1, 2, and 3 h after UV irradiation. Cytosol (C) and membranes (M) were subsequently separated using ultracentrifugation. Western blotting analysis on the different fractions confirmed that UpsX-HA migrates with the expected size of around 79 kDa on SDS-PAGE, with some smaller degradation products present in the cytosol fractions (Fig. 8). The 55 kDa protein was confirmed to be unspecific as it was also present in the negative control (−, lysate of MW001). Therefore, it was used as an internal loading standard. After UV induction, a clear increase in UpsX protein in line with the transcriptional response could be observed (Fig. 3). Interestingly, without UV induction, UpsX was found only in the cytosol while after UV stress, a significant fraction of about 35% of the protein seemed to localize to the membrane (Fig. 8).

## Discussion

T4P have shown to be involved in numerous functions both in bacteria as well as in archaea. In Sulfolobales, different type IV pili have been described, their functions include: motility, attachment to surfaces, biofilm formation, and DNA exchange. In *S. acidocaldarius,* three different T4P involved in these functions can be found: archaeal adhesive (aap) pili (Henche et al. 2012), archaella (Jarrell and Albers 2012), and ups pili (Fröls et al. 2008; Ajon et al. 2011). Previous studies have shown the involvement of ups pili in UV-induced cellular aggregation and DNA exchange. This mechanism is unique to the Sulfolobales and is proposed to be involved in repair of DNA DSBs (Fröls et al. 2008; Ajon et al. 2011). Here, we have studied the individual genes of the *S. acidocaldarius ups* gene cluster in more detail using bioinformatics, transcriptional analyses, deletion mutant analyses, and localization experiments.

Synteny analysis revealed that all Sulfolobales, but no other species contain a *ups* cluster encoding: UpsX, a protein with unknown function; UpsE, a secretion ATPase; UpsF, a membrane protein; and UpsA and B, two class III signal peptide-containing pilin subunits. Expression and induction of this operon has been shown for a number of *Sulfolobus* species (Ajon et al. 2011). The ups system of *A. hospitalis* is almost certainly not active as it lacks both pilin subunits and part of the *upsF* gene*,* suggesting that it lost these genes during evolution. A possible explanation of this loss might be that *Acidianus* species do not encounter as much UV light as other Sulfolobales and therefore have slower mutation rates. For instance, *A. hospitalis* is a facultative anaerobe (unlike obligate aerobic *Sulfolobus* and *Metallosphaera* species), may thus grow in deeper, darker, areas of hot springs (Giaveno et al. 2013) being less exposed to UV light. In other Sulfolobales*,* the *ups* genes can be readily deleted with no apparent effect on vegetative growth. However, the presence of ups pili does have a clear beneficial effect on the vitality of *Sulfolbus* cells upon severe DNA damaging conditions (Ajon et al. 2011). The ups system might therefore have evolved to conquer extremely DNA damaging conditions such as high temperatures, low pH values, and high UV doses. The increased exchange of DNA would make regular homologous recombination more efficient by increasing the chances of having a homologous template.

Operon mapping confirmed that the *ups* cluster of *S. acidocaldarius* is transcribed as one transcript demonstrating that it is truly an operon. Indeed, a deletion of the primary promoter region in front of *upsX* aborted pili formation, whereas a clean deletion of *upsX* has no effect. Deep sequencing data, moreover, showed the presence of additional TSSs within the operon in front of *upsE* and *upsA* (O. Wurtzel, unpubl. data). Secondary promoter elements were also found in the *S. acidocaldarius* archaella operon (Lassak et al. 2012) and might be essential to fine tune the stoichiometry of the different proteins within the ups pili: for example, many more pilin subunits will be needed than membrane proteins involved in the assembly of a functional pilus. The strong upregulation of the *ups* operon upon UV light was confirmed with qRT-PCR.

Deletion mutants of genes in the *ups* operon revealed that *upsE* and *upsF* are individually essential for pili formation as well as aggregation further expanding on the observation with the *upsEF* double-deletion mutant (Ajon et al. 2011). Deletion mutants of the pilin subunit genes *upsA* and *upsB* no longer showed UV-induced aggregation. Interestingly though, both strains still formed ups pili, but in lower numbers. These results suggest that UpsA and UpsB are both major pilin subunits that in wild-type cells might form a mixed pilus structure, this in contrast to bacterial T4P systems which were so far found to have only one major pilin subunit (Ayers et al. 2010; Giltner et al. 2012). In bacteria, minor and major pilins have been implicated in adherence to various different surfaces, cells from the same species, and host tissues (reviewed by Giltner et al. 2012). Also ups subunits are thought to be involved in strain-specific recognition and interaction between cells from the same species (Ajon et al. 2011). Preliminary data suggest that a specific region in the pilin subunit UpsA is responsible for the recognition of glycosylated S-layer proteins from cells from the same species (M. van Wolferen and S.-V. Albers, unpubl. data).

UpsX is a protein with so far unknown function, but it is highly conserved in all species that contain a *ups* operon suggesting that it fulfills an essential role in UV-dependent DNA transfer. Intriguingly, however, a deletion mutant of *upsX* still formed ups pili, and the UV-induced cellular aggregation was comparable to wild type. UpsX therefore does not seem to play a structural role in the ups pilus nor is it essential for cellular recognition or assembly of the pili. Possibly other functions of UpsX would be DNA transfer, and indeed, a UV-induced *upsX* deletion mutant contributed less to DNA transfer compared to the wild-type strain. These data indicate that UpsX promotes DNA exchange but it is not essential for this process. Interestingly, a significantly larger portion of the UpsX protein localized to the membrane fraction in time upon UV exposure. Possibly, UpsX promotes DNA exchange at the membrane via a yet unknown mechanism. Future biochemical experiments might provide more insights into the actual function of UpsX.

## Supporting Information

**Figure S1.** Alignment of UpsX from different Sulfolobales; *Acidianus hospitalis (Ahos), Metallosphaera cuprina (Mcup), Metallosphaera sedula (Msed), Metallosphaera yellowstonensis (Myel), Sulfolobus tokodaii (ST), Sulfolobus acidocaldarius (Saci), Sulfolobus solfataricus (Sso),* and *Stygiolobus azoricus (Staz)*.

**Table S1.** Primers used during this study.
