# Corrigendum

**DOI:** 10.1002/mbo3.426

**Published:** 2016-12-08

***MicrobiologyOpen*** **2013; 2(6): 928‐937**

Molecular analysis of the UV‐inducible pili operon from *Sulfolobus acidocaldarius*

Marleen van Wolferen, Małgorzata Ajon, Arnold J. M. Driessen & Sonja‐Verena Albers

10.1002/mbo3.128

Correction regarding *Sulfolobus acidocaldarius* strain GA06

Upon resequencing we discovered that strain GA06 was not, as published (van Wolferen et al., 2013), a deletion of the *upsX* ORF and a 40 bp upstream region, but was a deletion starting 55 bp upstream and ending 35 bp downstream of *upsX*. Since this could affect the genes downstream of UpsX, a clean deletion mutant of the *upsX* promoter (all 261 bp upstream of *upsX*) was created and analysed by qRT‐PCR studies as well as by UV‐induced aggregation assays. qRT‐PCR experiments showed that in this strain, *upsE, F, A* and *B* were upregulated normally upon UV‐induction, and aggregation experiments showed that the deletion mutant of the *upsX* promoter aggregated in a similar manner as the WT strain after treatment with UV‐light. These results are similar to the results obtained for the deletion of the *upsX* gene (MW115, Δ*upsX,* Figures 4 and 5). Therefore, the promoter in front of *upsX* is not the primary promoter of the *ups‐*operon and is also not essential for transcription of the other *ups‐*genes.

Additionally, we discovered that the EM image in Figure 6 (GA06) was accidently replaced by an image of strain (*ΔupsEF*). Both strains did not show any UV induced pili, but the specific image shown was not obtained for this strain. All other strains and EM pictures and the conclusions obtained with these strains are correct.

