# Supplementary material for: Molecular analysis of the UV-inducible pili operon from Sulfolobus acidocaldarius
Source: Microbiologyopen. 2013 Sep 19;2(6):928–37. doi: 10.1002/mbo3.128 (PMC3892339; doi:10.1002/mbo3.128)
Supplement: Supplementary file 1 [file mbo30002-0928-SD1.docx]

**Supplementary Material to “Molecular analysis of the UV-inducible pili operon from *Sulfolobus acidocaldarius”***

By Marleen van Wolferen, Małgorzata Ajon, Arnold J. M. Driessen and Sonja-Verena Albers

**
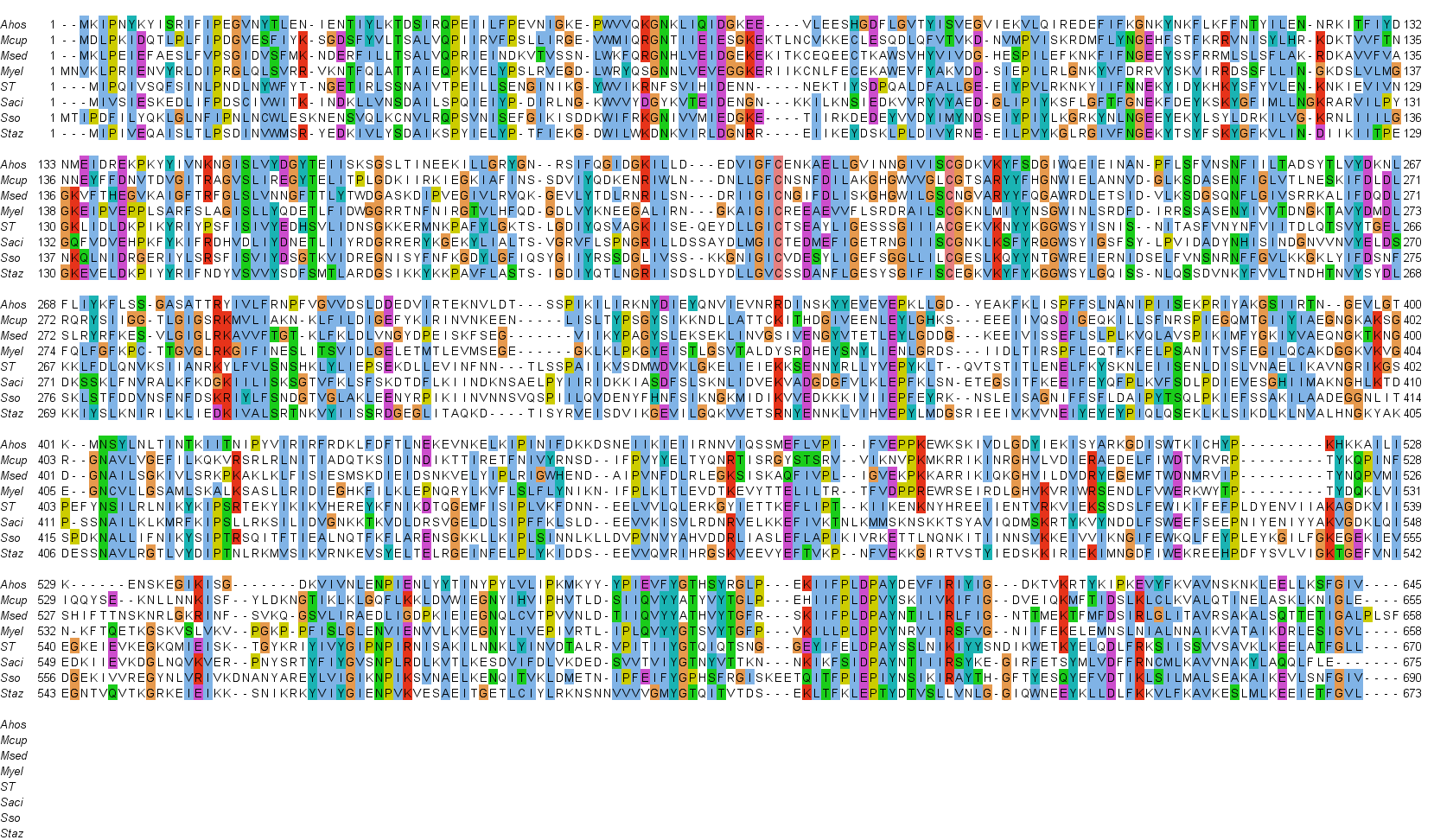
**

**Figure S1:** Alignment of UpsX from different Sulfolobales; *Acidianus hospitalis (Ahos), Metallosphaera cuprina (Mcup), Metallosphaera sedula (Msed), Metallosphaera yellowstonensis (Myel), Sulfolobus tokodaii (ST), Sulfolobus acidocaldarius (Saci), Sulfolobus solfataricus (Sso), Stygiolobus azoricus (Staz).*

**Table S1: Primers used during this study**

| **Primer** | **Sequence** | **Purpose** |
| --- | --- | --- |
| 19 | CCCGCGGATCCCGAGATAGGCAATAGTAATATGG | Δ*upsX* -40_US_RV_BamHI |
| 20 | CGCGCCGCGGCATTTAATCCTGCATAGCCTGAG | Δ*upsX* -40_US_FW_SacII |
| 21 | GCGGCCCGGGCTTATGGCATGGATCAGGTATTAG | Δ*upsX* -40_DS_FW_SmaI |
| 22 | GCCCTGCAGCCGTGAAATGTGGTGATGGAAC | Δ*upsX* -40_DS_RV_PstI |
| 546 | CGCGCTGCAGCCTGCATAGCCTGAGCCTACTATAATCG | Δ*upsX*_US_FW_ApaI |
| 547 | GCTAATACCTGATCCATTTATTTCTTCGATGCTGTAAAATATAC | Δ*upsX*_US_RW |
| 548 | CAGCATCGAAGAAATAAATGGATCAGGTATTAGCAGAG | Δ*upsX*_DS_FW |
| 549 | CGCGCGGATCCGTGAAATGTGGTGATGGAACCGTG | Δ*upsX*_DS_RV_BamHI |
| 2000 | GTAGGGCCCCCAGTTAGTTAAGCTTTTACCAG | Δ*upsA*_US_FW_ApaI |
| 2001 | GAGATTCCTTTCATCTTTCCTCAAATAAAATGAATC | Δ*upsA*_US_RW |
| 2002 | TTGAGGAAAGATGAAAGGAATCTCTTCAATTTTTTC | Δ*upsA*_DS_FW |
| 2003 | GCGGATCCGCAATCAAAGCCGACTTGTCTG | Δ*upsA*_DS_RV_BamHI |
| 2004 | GAGGGCCCTCTATTTCCCAACAATTCTAATG | Δ*upsB*_US_FW_ApaI |
| 2005 | GAATAGAATAGTCCTAATTACACCGTAGAAGCTAG | Δ*upsB*_US_RW |
| 2006 | CGGTGTAATTAGGACTATTCTATTCTTTTTTAG | Δ*upsB*_DS_FW |
| 2007 | GCGGATCCAAACCACATCAGCTGTCTTATCAC | Δ*upsB*_DS_RV_BamHI |
| 2008 | GAATAGAATAGTTTTAACCAACTTTCCTCAAATAAAATG | Δ*upsAB*_US_RW |
| 2009 | GAAAGTTGGTTAAAACTATTCTATTCTTTTTTAG | Δ*upsAB*_DS_FW |
| 2010 | GTAGGGCCCGTGTATAATGATGACCTATTTAGCTG | Δ*upsE*_US_FW_ApaI |
| 2011 | CTAATATTTTCAAGCCATAAGAAGGAAATATTAAAAG | Δ*upsE*_US_RV |
| 2012 | CTTCTTATGGCTTGAAAATATTAGCATGTGATATATTC | Δ*upsE*_DS_FW |
| 2013 | GTCGGATCCCTTAATCTATCCTTAAGCGAAACGC | Δ*upsE*_DS_RW_BamHI |
| 2014 | AAGGGATAATAGAGTAGAAC | Δ*upsE*_Check_FW |
| 2015 | GTAAACTGGAAGCCTATAAGG | Δ*upsE*_Check_RV |
| 2016 | GTAGGGCCCGATAATAGGTGAGGTAAGAGG | Δ*upsF*_US_FW_ApaI |
| 2017 | AAATGAATCTTTTAATCACATGCTAATATTTTC | Δ*upsF*_US_RV |
| 2018 | AGCATGTGATTAAAAGATTCATTTTATTTG | Δ*upsF*_DS_FW |
| 2019 | GTCGGATCCGAGATTCCTTTCATCCTAATTAC | Δ*upsF*_DS_RW_BamHI |
| 2020 | GGTTAGATTTATAGCAAGATCAAC | Δ*upsF*_Check_FW |
| 2021 | GTATTCATAAGAGTTGGATAGCG | Δ*upsF*_Check_RV |
| 2028 | CTTCTCCTAAATGTTAATCTG | Δ*upsA*_Check_FW |
| 2029 | GTTGGATAGCGAATTGGTCG | Δ*upsA*_Check_RV |
| 2030 | ACTTCAAAGATGATTGCAGGAG | Δ*upsB*_Check_FW |
| 2031 | TTTCCACATATGTACGATGAG | Δ*upsB*_Check_RV |
| 2073 | AATTTAGCATAGACCCAGCTTAC | qPCR *upsX* fw |
| 2074 | ATTTACTACTGCCTTCAGCATAC | qPCR *upsX* rv |
| 2075 | GCTAGTAAAGCCAACAAGAGTG | qPCR *upsE* fw |
| 2076 | ATATAGTCGCTGCTACCCTATG | qPCR *upsE* rv |
| 2077 | TAGAGGAGCTAGCAGGAACAC | qPCR *upsF* fw |
| 2078 | ACAACATGACCGGAGTCAG | qPCR *upsF* rv |
| 2079 | TAGCCAGGGTATGTTCAGTAATC | qPCR *upsA* fw |
| 2080 | ACCTAAGTTCCCGTTATTGAC | qPCR *upsA* rv |
| 2081 | GACCAATTCGCTATCCAACTC | qPCR *upsB* fw |
| 2082 | CTGCATGTCTGATTTCCTACC | qPCR *upsB* rv |
| 3030 | GTTACGCGTAGTCCGGAACGTCATACGGGTAGGAG-CCTTCAAGGAATAATTGCTGTGCTAAG | *upsX*-HA_US_rv |
| 3031 | GAAGGCTCCTACCCGTATGACGTTCCGGACTACGCG-TAACTTTCATTTTTCTGTCTGTC | *upsX*-HA_DS_fw |
|  |  |  |
|  |  |  |
|  |  |  |
|  |  |  |
